# Two Sides of the Same Coin: Cannabis Dependence and Mental Health Problems in Help-Seeking Adolescent and Young Adult Outpatients

**DOI:** 10.1007/s11469-012-9378-1

**Published:** 2012-03-28

**Authors:** Melissa M. Norberg, Robert A. Battisti, Jan Copeland, Daniel F. Hermens, Ian B. Hickie

**Affiliations:** 1 National Cannabis Prevention and Information Centre, University of New South Wales, PO Box 684, Randwick, NSW 2031 Australia; 2 Clinical Research Unit, Brain & Mind Research Institute, University of Sydney, Camperdown, Australia

**Keywords:** Cannabis use, Cannabis dependence, Comorbidity, Anxiety, Mood disorders, Young people

## Abstract

The aim of the current study was to delineate the psychiatric profile of cannabis dependent young people (14–29 years old) with mental health problems (*N* = 36) seeking treatment via a research study. To do so, the Structured Clinical Interview for DSM-IV-TR Axis I Disorders and the Structured Clinical Interview for DSM-IV Childhood Diagnoses were used to obtain DSM-IV diagnoses, while a modified Timeline Followback interview and self-reports were used to measure cannabis use, cannabis-related problems, and impairment. Most individuals had at least two Axis I disorders in addition to cannabis dependence. Anxiety disorders were common, with posttraumatic stress disorder, social phobia, and generalised anxiety disorder accounting for the majority of these diagnoses. On average, young people reported a moderate degree of dependence and functional impairment, and a substantial number of cannabis-related problems. Although both males and females reported using similar quantities of cannabis per month, females reported using cannabis more frequently than males. The current data suggest that young people who present for cannabis use treatment in the context of a mental health issue may have a variety of psychiatric problems that need addressed and that males and females may have slightly different profiles. If cannabis use treatments are to advance for this population, more attention needs to be paid to the complex issues that young people present to treatment with.

Although cannabis is the most common cause of illicit drug treatment admissions in the USA and Australia (AIHW 2008, 2010; SAMHSA 2008; UNODC 2007), only one in three dependent individuals seek treatment, (Compton et al. 2007; Stinson et al. 2006) with young people being particularly unlikely to seek treatment (Kessler et al. 2001; Schmidt 2007). Highlighting the disparity between suitability and desire for treatment, the USA Cannabis Youth Treatment Study found that while 96 % of participants met abuse or dependence criteria, only 20 % believed they had a problem (Diamond et al. 2006). Recent research has discovered that receiving treatment for a co-occurring psychiatric disorder increases the chance that someone will seek treatment for cannabis dependence (Agosti and Levin 2007; Arendt et al. 2007; Strike et al. 2003).

Although epidemiological studies suggest a history of psychiatric problems is associated with seeking treatment for cannabis use the majority of cannabis treatment outcome studies have inferred mental health issues from self-report measures (e.g., Copeland et al. 2001; Rigter et al. 2010; Stephens et al. 2002). Only three cannabis specific treatment trials have reported on the rates of psychiatric comorbidity in their samples using diagnostic interviews. In the first study, Dennis and colleagues (Dennis et al. 2004) reported on the clinical profile of adolescents (most involved with the criminal justice system) who received one of five different short-term outpatient interventions for cannabis. Ninety-five percent of this sample reported at least one other comorbid problem, with conduct disorder (53 %), attention deficit-hyperactivity disorder (38 %), and alcohol use disorders (37 %) being the most common. Internalising disorders were less common, and rates were reported only for major depression (18 %), generalised anxiety disorder (23 %), and traumatic stress disorders (14 %). Unsurprisingly, Kamon and colleagues (Kamon et al. 2005) found a similar clinical profile for externalising disorders in adolescents who participated in a contingency management intervention for cannabis abuse and conduct problems. Unfortunately, specific internalising disorders were not specified. In the third study, Carroll and colleagues (Carroll et al. 2006) reported on the prevalence of lifetime co-occurring disorders for cannabis-dependent young adults who were referred by the criminal justice system for contingency management and motivational/skill-building therapy for cannabis dependence. A quarter of the sample met criteria for an alcohol use disorder, 11 % met criteria for a depressive disorder, and 22 % met criteria for an anxiety disorder. Again, specific anxiety and depressive disorders were not reported nor the extent of comorbidity. Taken together, these data suggest that research participants are likely to have a variety of concurrent Axis I disorders; however, information as to the specific types and extent of comorbidity is limited, particularly so for young people not involved with the criminal justice system.

The aim of the current study was to address the limitations of the existing clinical profile data by reporting on the extent of co-occurring mental health disorders in young adults presenting for inclusion in a research study that examined the efficacy of cannabis use treatment for individuals with a co-occurring mental health issue. Diagnoses obtained from a semi-structured clinical interview were the focus of the present study as self-report instruments are limited to problem severity and greatly limit clinical generalisability. Cannabis use diagnoses were supplemented with data on cannabis use and cannabis-related problems and impairment to provide an in depth analysis of the unique features of the young people’s use and to facilitate comparisons with published cannabis treatment research. Peters et al. (2011) found that the majority of published randomized controlled trials for cannabis use disorders included measures of cannabis use and associated problems. Given that gender differences exist in patterns of mental illness and help-seeking behaviour (Cuffe et al. 1995; Nolen-Hoeksema and Girgus 1994; Wittchen et al. 1998), data are presented separately for young men and women. An age span of 14 to 29 years was chosen given that epidemiological studies have found this group to be at greatest risk for developing comorbid mental health and substance use disorders (Wittchen et al. 2007; Wittchen, et al. 1998). The current clinical profile data will assist researchers and clinicians in understanding young people who participate in clinical research studies for cannabis use via outpatient based mental health centers.

## Method

### Participants

Thirty-six individuals presented for a baseline assessment for a pilot study examining a brief intervention for cannabis use for young individuals with co-occurring Axis I disorders. (One individual did not complete the structured clinical interview due to a loss of interest in completing the study.) Participants were recruited via (i) flyers placed within community mental health settings, (ii) advertisements placed within popular music magazines, and (iii) mental health professionals working within the specialised mental health outpatient service where the research study was conducted (n.b., the service specialised in a range of disorders other than drug and alcohol disorders). In this latter case, mental health professionals notified clients they were treating for a non-substance use problem about the study. In this way, help-seeking young people in the current study were participants interested in obtaining treatment for cannabis use, whether that interest was self-initiated or initiated by a mental health professional.

### Measures

A brief interview developed for the purposes of this study assessed demographic characteristics, cannabis use history, concurrent substance use, and potential mental health problems. The Structured Clinical Interview for DSM-IV-TR Axis I Disorders: Research Version (SCID-RV; First et al. 2002) was used to determine clinical diagnoses. Following the SCID-RV instructions, severity of cannabis dependence was based on functional impact during the last month. As such, diagnoses of mild severity were those with few symptoms in excess of what was required to specify a diagnosis and the symptoms resulted in no more than mild impairment. Moderate severity diagnoses were those with symptoms or functional impairment in the moderate range, whereas severe diagnoses were those with many symptoms in excess of what was required to specify a diagnosis and the symptoms markedly interfered with functioning (e.g., unemployment, homelessness, family estrangement). Primary versus secondary diagnoses were based on clinical judgment regarding which disorder was currently causing the most impairment. Additionally, the Structured Clinical Interview for DSM-IV Childhood Diagnoses (KID-SCID; Hien et al. 2004) was used to assess attention deficit-hyperactivity disorder, oppositional defiant disorder, and conduct disorder in individuals under the age of 18 (*n* =3). The SCID-RV screening module (revised to include initial assessment of childhood disorders) was used to determine appropriate SCID modules to administer.

The Timeline Followback interview (TLFB; Sobell and Sobell 1996) was used to assess frequency and quantity of cannabis used during the past 90 days. The TLFB was modified to improve quantity assessment. Each individual was asked to replicate their standard joint or cone using a cannabis substitute (marijuanilla), which was then weighed (Norberg et al. 2012). Separate weights were obtained for cannabis and mixed-in tobacco. Daily use was obtained by querying each individual about the number of joints or cones they had on each use day and multiplying this number by each weight. Typical patterns of use facilitated recall on the TLFB. Severity and impact of cannabis use were assessed using the Severity of Dependence Scale (SDS; Gossop et al. 1997; Martin et al. 2006a), the Adolescent Cannabis Problems Questionnaire (CPQ; Martin et al. 2006b), and the Sheehan Disability Scale (SHDS; Sheehan 1986). All instruments have adequate to excellent psychometric properties.

### Procedure

In order to be invited to the baseline assessment, participants had to report during a screening interview that they were between 14 and 29 years old, fluent in English, had a mental health problem, and used cannabis at least weekly during the last month. Written informed consent was obtained prior to completing the baseline assessment. A research assistant reimbursed participants with $AU50 in vouchers after the baseline assessment for their time and travel expenses. Interviews were conducted by a doctoral student in clinical psychology, who underwent training and supervision in the SCID-RV, KID-SCID, and TLFB. All SCID-RV and KID-SCID diagnoses were reviewed and agreed upon by the first author, an experienced clinical psychologist with expertise in co-occurring mental health and substance use disorders. This study was approved by the Human Research Ethics Committees at the University of New South Wales and at the University of Sydney. The clinical trial protocol was lodged with the Australian and New Zealand Clinical Trials Registry (ACTRN12611000169943).

### Statistical Analyses

A *t*-test and a series of Pearson’s chi-square tests of independence were conducted to examine potential differences between males and females on demographic variables. Comparisons between males and females on cannabis use (estimated via measurement of the cannabis substitute) and resulting impact were made using analysis of variance (ANOVA). TLFB cannabis use variables were log transformed prior to conducting inferential analyses as they were skewed positively. McNemar’s test of dependent proportions examined if certain diagnostic categories (anxiety, mood, childhood, substance use, and psychotic disorders) were more prevalent than others in the current sample (Conover 1999). Pearson’s chi-square tests examined whether diagnostic categories differed between males and females.

## Results

### Sample Characteristics

Demographic details are presented in Table 1. Males were statistically significantly more likely to smoke cigarettes and use hallucinogens than females. There was a marginally significant difference between males and females for living situation. Males were somewhat more likely to live with family members, while females were somewhat more likely to live with friends. Table 2 displays information about cannabis use, cannabis-related problems, and functional impairment. Females used cannabis 7 days more per month on average than males, despite comparable amount of cannabis use per month. Males and females did not statistically differ on any other variables.

**Table 1:** Demographics

Variable	Males (*n* = 18)	Females (*n* = 18)	*p*
Age	23.67	22.22	0.21
95 % *CI*	22.06–25.28	20.61–23.83	
Australian born	17 (94.4 %)	13 (72.2 %)	0.07
ATSI	1 (5.6 %)	1 (5.6 %)	1.00
Living situation			0.05
Alone	2 (11.1 %)	3 (16.7 %)	
Family	14 (77.8 %)	7 (38.9 %)	
Friends	2 (11.1 %)	8 (44.4 %)	
Current legal problems	2 (11.1 %)	3 (16.7 %)	0.63
Employed/studying	10 (55.6 %)	14 (77.8 %)	0.16
Quit school	9 (50.0 %)	7 (38.9 %)	0.50
Dating/married	7 (38.9 %)	8 (44.4 %)	0.74
Current cigarette smoker	17 (94.4 %)	11 (61.1 %)	0.02
Current alcohol drinker	16 (88.9 %)	17 (94.4 %)	0.55
Current ecstasy user	7 (38.9 %)	6 (33.3 %)	0.73
Current hallucinogen user	8 (44.4 %)	1 (5.6 %)	0.01
Current amphetamine user	5 (27.8 %)	2 (11.1 %)	0.21

*ATSI* Aboriginal or Torres Strait Islander

**Table 2:** Cannabis use, cannabis-related problems, and functional impairment

Variable	Group	*N*	*M*	*95 % CI*	*F*	*df*	*p*	$n_p^2$
Grams of cannabis used per month	Males	18	39.29	19.05–81.04	0.00	1,34	0.99	0.00
	Females	18	39.24	19.01–80.96				
Days of use per month	Males	18	19.16	15.69–23.41	4.94	1,34	0.03	0.13
	Females	18	26.10	21.37–31.88				
Grams of tobacco used per month	Males	18	16.81	7.11–38.13	0.11	1,34	0.74	0.00
	Females	18	20.43	8.76–46.09				
SDS	Males	18	7.72	6.03–9.41	0.90	1,34	0.35	0.03
	Females	18	8.83	7.15–10.52				
CPQ	Males	18	18.33	16.42–20.24	0.39	1,34	0.54	0.01
	Females	18	17.50	15.59–19.41				
SHDS-school/work	Males	18	4.89	3.43–6.35	0.00	1,34	0.96	0.00
	Females	18	4.83	3.38–6.29				
SHDS-social life	Males	18	5.61	4.31–6.92	2.73	1,34	0.11	0.07
	Females	18	4.11	2.81–5.42				
SHDS-family life	Males	18	5.83	4.40–7.27	1.00	1,34	0.32	0.03
	Females	18	4.83	3.40–6.27				

Grams of cannabis and tobacco were based on weights on substitute cannabis (marijuanilla). *SDS* Severity of Dependence Scale; *CPQ* Cannabis Problems Questionnaire; *SHDS* Sheehan Disability Scale. $n_p^2$ is a measure of effect size, which indicates how much of the variance of the dependent variable was associated with the independent variable

### Cannabis Dependence

Based on the SCID-RV, two patients (one male, one female; 5.7 %) met criteria for cannabis dependence of mild severity, 18 (8 males, 10 females; 51.4 %) met criteria for moderate severity, and 15 (9 males, 6 females; 42.9 %) were classified as severely dependent. Interestingly, cannabis dependence was the primary disorder for only four (1 male, 3 females; 12.1 %) of the 33 individuals with co-occurring disorders*.*

### Prevalence of Comorbidity

Table 3 displays diagnostic information, separated by biological sex. Two of the 35 individuals who completed the structured clinical interviews did not meet full criteria for a non-substance use disorder. Thirty-three participants (94.3 %) had two or more Axis I disorders, twenty-six participants (74.3 %) had three or more Axis I disorders, and five participants (14.3 %) had four Axis I disorders. Table 3 displays the distributions of disorders across biological sex. Young people were statistically more likely to be diagnosed with anxiety disorders than psychotic disorders (*p* = 0.001), childhood disorders (*p* < 0.001), and substance use disorders other than cannabis (*p* = 0.005). Young people also were statistically more likely to be diagnosed with mood disorders than childhood disorders (*p* < 0.001) and substance use disorders other than cannabis (*p* = 0.01). Young people were marginally more likely to be diagnosed with mood disorders (*p* = 0.05) than psychotic disorders. Psychiatric disorder proportions did not statistically significantly differ between males and females (*p* > 0.13 in all cases).

**Table 3:** Distribution of disorders

Disorder	Males *(n* = 18)		Females (*n* = 17)		Total (*n* = 35)	
	Count	%	Count	%	Count	%
Cannabis dependence	18	100 %	17	100 %	35	100 %
Other substance use disorders	4	22.2 %	4	23.5 %	8	22.9 %
Alcohol abuse	1	5.6 %	1	5.9 %	2	5.7 %
Alcohol dependence	3	16.7 %	2	11.8 %	5	14.3 %
Hallucinogen dependence	0	0 %	1	5.9 %	1	2.9 %
Mood disorders	12	66.7 %	7	41.2 %	19	54.3 %
Bipolar I	1	5.6 %	1	5.9 %	2	5.7 %
Bipolar II	1	5.6 %	0	0 %	1	2.9 %
Major depressive disorder	7	38.9 %	4	23.5 %	11	31.4 %
Dysthymic disorder	3	16.7 %	0	0 %	3	8.6 %
Depressive disorder NOS	0	0 %	1	5.9 %	1	2.9 %
Substance-induced mood disorder	0	0 %	1	5.9 %	1	2.9 %
Psychotic disorders	5	27.8 %	4	23.5 %	9	25.7 %
Schizophrenia	3	16.7 %	1	5.9 %	4	11.4 %
Schizoaffective disorder	1	5.6 %	1	5.9 %	2	5.7 %
Substance induced psychotic disorder	0	0 %	1	5.9 %	1	2.9 %
Psychotic disorder NOS	1	5.6 %	1	5.9 %	2	5.7 %
Anxiety disorders	11	61.1 %	13	76.5 %	24	68.6 %
Social phobia	7	38.9 %	1	5.9 %	8	22.9 %
Posttraumatic stress disorder	3	16.7 %	7	41.2 %	10	28.6 %
Generalized anxiety disorder	2	11.1 %	5	29.4 %	7	20.0 %
Substance induced anxiety disorder	0	0 %	1	5.9 %	1	2.9 %
Anxiety disorder NOS	0	0 %	1	5.9 %	1	2.9 %
Childhood disorders	0	0 %	1	5.9 %	1	2.9 %
ADHD	0	0 %	1	5.9 %	1	2.9 %

Data for diagnostic categories reflect how many individuals had at least one disorder of that type; whereas data for specific disorders reflect how many people were diagnosed with that actual disorder. Thus, the number of specific diagnoses are greater than their corresponding categories because most participants were diagnosed with more than one disorder. *NOS* Not Otherwise Specified

## Discussion

Although limited by the small sample size, this is the first comprehensive diagnostic assessment of young people seeking treatment for cannabis dependence via a research study. Approximately 43 % of the sample met criteria for severe cannabis dependence based on the SCID-RV. In regards to psychiatric problems, the young people met criteria for a diverse array of concurrent psychiatric diagnoses and most had at least two Axis I disorders in addition to cannabis dependence. Anxiety and mood disorders were more common than psychotic disorders, childhood disorders, and substance use disorders other than cannabis. Thus, internalising disorders were more prevalent and externalising disorders were less prevalent in the current study than in past cannabis use treatment studies (Carroll, et al. 2006; Dennis, et al. 2004; Kamon, et al. 2005). This difference may have occurred for two reasons. First, previous studies have restricted their samples to adolescents and have primarily recruited individuals involved with the criminal justice system or who had conduct disorder. Second, the present study only examined disorders usually first diagnosed in childhood in individuals who were under the age of 18 (*n* = 3). Although it is uncommon to assess these disorders in individuals over the age of 18, it means that these disorders were examined in only 8.6 % of the sample. Despite having a different psychiatric profile from past treatment research, the diagnostic composition of the current sample is unsurprising given research on treatment seeking behaviour. Using data from the Australian National Survey of Mental Health and Well-Being (NSMHW), Degenhardt and colleagues (Degenhardt et al. 2001) demonstrated that the presence of an anxiety disorder or mood disorder was the largest determinant of seeking treatment for any problem, regardless of level of involvement with cannabis use. Approximately three out of five cannabis dependent individuals in the NSMHW who had a mood disorder had sought treatment, whereas four out of every five individuals who had an anxiety disorder had sought treatment. Although not statistically significant, in the current study males were marginally more likely to be diagnosed with a mood disorder than were females. In addition, examination of the specific anxiety disorders demonstrated that cannabis dependent young males might be more likely to present with social anxiety, while females may be more likely to present with trauma issues. When aggregating anxiety and depression together, previous research found that young women were more likely to experience anxiety and depression than young men (Patton et al. 2002). Thus, the current findings highlight that future research should not limit itself to broad diagnostic categories as different psychiatric profiles may emerge when examining individual disorders and biological sex separately.

Participants reported experiencing a substantial number of cannabis-related problems and perceived these problems to result in moderate functional impairment across their school/work, social, and family life. Although males and females reported using the same amount of cannabis per month, females reported using approximately 7 days more per month than males. Thus, females smoked less cannabis per day of use than males. Related, males evidenced a tendency to meet criteria for a severe diagnosis of cannabis dependence, whereas females were somewhat more likely to have symptoms of a moderate severity. Previous research with larger sample sizes has shown gender not to differentiate moderate (at least once a month, but less than once a week) users from heavy (at least once a week) users of cannabis (Barnes et al. 2005), but has shown that young males are more likely to meet criteria for cannabis dependence than are females (Cotto et al. 2010). Given the current findings and findings from past research, it may be that quantity of cannabis per use episode is a better determinant of dependence than frequency of use. As such, quantity of use could explain the differences between males and females in published rates of cannabis dependence in epidemiological samples. Based on this hypothesis, future studies would benefit from assessing both frequency and quantity of use.

Noteworthy, cannabis dependence was the primary disorder for only 12.1 % of the sample with co-occurring disorders. Although important, this finding may be a sampling bias of the clinic where individuals sought help. Individuals who believe their psychiatric issues are more problematic than their substance use may be particularly inclined to seek a specialised mental health service for help. At the same time, this finding may indicate that help-seeking young persons may be trying to cope with their psychiatric problems by using cannabis. If true, treatments that only target the mental health issue or only target the cannabis use may fail to address young people’s needs. Empirical studies are needed to assess the relative efficacy of sole-focused treatments, concurrent provision of mental health and substance use treatments, and integrated treatment that targets the interrelated nature of mental health problems and substance use. There is a paucity of research in this area (Baker et al. 2010; Lubman et al. 2010), outcome data from the current sample suggest that a brief intervention focused on developing alternative strategies to using cannabis to cope with a mental illness is not substantial enough to considerably improve cannabis use or mental health symptoms (Norberg et al. 2011).

Limitations of this study include the small sample size and multiple statistical tests. As such, it is not surprising that few statistical differences were noted between males and females. This study should not been interpreted as evidence that differences rarely exist between young male and female cannabis users. Rather the study should be viewed as it was intended at conception—as a feasibility study to demonstrate that cannabis users are willing to participate in a comprehensive assessment as part of a treatment study and as a basis to generate hypotheses for larger treatment outcome studies. Future research with larger samples would benefit from assessing the reliability and validity of the TLFB and quantity/frequency indices for assessing cannabis use in comparison to and urine tests. Use of a cannabis substitute signifies that the reported grams of use in the current study may only be valid for comparisons within this sample. Although likely unrealistic, future research would benefit from participants providing samples of their own cannabis for weight and volumetric comparisons with the cannabis substitute. A further limitation includes the recruitment process. It is not clear how well the profiles presented in this study will generalize to patients seeking treatment via research studies not conducted at a specialised mental health service. Finally, all participants had a diagnosis of cannabis dependence; psychiatric profiles may differ for those with a cannabis abuse diagnosis.

The strengths of this study are as follows. First, the study describes disorders based on a semi-structured clinical interview conducted by a trained clinician, whereas most previous studies have relied upon self-report measures such as the Symptom Checklist-90, Beck Depression Inventory, or State-Trait Anxiety Inventory. These measures provide only an indication of psychological distress. In addition, past studies typically have focused on externalizing disorders or diagnostic categories (e.g. mood or anxiety disorders) rather than the clinically important detailed diagnoses provided in this study. Third, this study did not exclude individuals with psychotic disorders, nor was it exclusively focused on individuals with psychotic disorders. Thus, the present study examined a broad spectrum of individuals with mental health difficulties who sought a brief treatment for their cannabis use, making it the first of its kind. Fourth, this study included individuals who were seeking treatment from a specialized youth mental health service instead of relying solely on media advertisements, which allowed for an assessment of the clinical profiles of young people that present to treatment, not just individuals who present for research studies. Finally, this study used a modified Timeline Followback approach that allowed for assessment of both cannabis and tobacco as 64.8 % of Australians report using cannabis mixed with tobacco (AIHW 2007).

In summary, the current data suggest that young people who present for cannabis use treatment in the context of a mental health issue may have a variety of psychiatric problems that need addressed. Males and females may have slightly different profiles. Thus, if cannabis use treatments are to advance for this population, more attention needs to be paid to the complex issues that young people present to treatment with.
